# Probing the Hidden Photoisomerization of a Symmetric Phosphaalkene Switch

**DOI:** 10.1002/anie.202419943

**Published:** 2024-12-13

**Authors:** Rajesh Deka, Jorn D. Steen, Michiel F. Hilbers, Wim G. Roeterdink, Alessandro Iagatti, Ruisheng Xiong, Wybren Jan Buma, Mariangela Di Donato, Andreas Orthaber, Stefano Crespi

**Affiliations:** ^1^ Department of Chemistry—Ångström Laboratory Uppsala University Box 523 751 20 Uppsala Sweden; ^2^ Van't Hoff Institute for Molecular Sciences University of Amsterdam Science Park 904 1098 XH Amsterdam The Netherlands; ^3^ Department of Chemistry - BMC Uppsala University Box 576 751 23 Uppsala Sweden; ^4^ Institute for Molecules and Materials, FELIX Laboratory Radboud University Toernooiveld 7c 6525 ED Nijmegen The Netherlands; ^5^ ICCOM-CNR via Madonna del Piano 10 50019 Sesto Fiorentino Italy; ^6^ Laboratorio Europeo di Spettroscopia Non Lineare (LENS) via N. Carrara 1 50019 Sesto Fiorentino Italy; ^7^ INO-CNR Largo Fermi 6 50125 Firenze Italy

**Keywords:** Phosphaalkene, Excited state dynamics, NMR spectroscopy, Photoisomerization, Laser spectroscopy

## Abstract

In this study, we present the synthesis and analysis of a novel, air‐stable, and solvent‐resistant phosphaalkene switch. Using this symmetric switch, we have demonstrated degenerate photoisomerization *experimentally* for the first time. With a combination of photochemical‐exchange NMR spectroscopy, ultrafast transient absorption spectroscopy, and quantum chemical calculations, we elucidate the isomerization mechanism of this symmetric phosphaalkene, comparing it to two other known molecules belonging to this class. Our findings highlight the critical role of the isolobal analogy between C=P and C=C bonds in governing nanoscale molecular motion and break new ground for our understanding of light‐induced molecular processes in symmetric heteroalkene systems.

## Graphical abstract

This study presents the synthesis and analysis of a novel, air‐stable, solvent‐resistant phosphaalkene switch exhibiting degenerate photoisomerization. The research elucidates the isomerization mechanism using PC‐EXSY NMR spectroscopy, ultrafast transient absorption spectroscopy, and MRSF‐TDDFT calculations. The work deepens our understanding of symmetric heteroalkene systems, opening new avenues for main group‐based molecular machines.

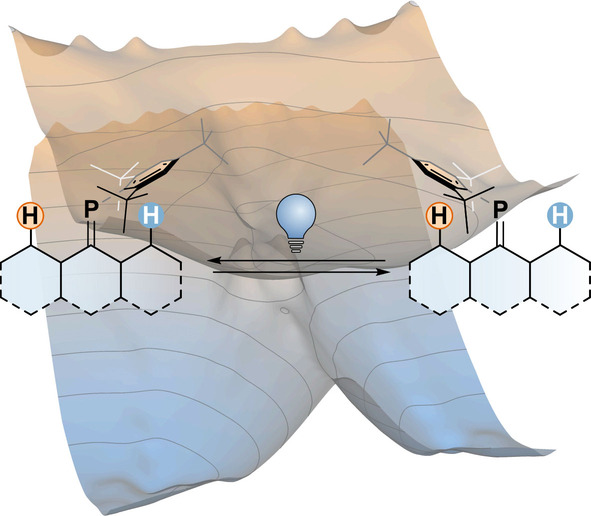

Molecular photoswitches are a class of molecules that undergo reversible photoisomerization allowing precise control of their geometry, polarity, rigidity, and electronic structure.[^1^, ^2^] After decades of fundamental and applied research, these systems have become a cornerstone of different interdisciplinary subjects. These research fields benefit from the possibility of achieving remarkable property modulation at different length scales, encompassing nano‐[^3^, ^4^, ^5^] and macroscopic effects^[6]^ that surpass simple color changes. In this way, recent years have witnessed multiple applications in smart materials,[^7^, ^8^, ^9^, ^10^] photobiology,^[11]^ imaging,[^12^, ^13^] catalysis,^[14]^ solar energy conversion,[^15^, ^16^] and xolography,^[17]^ to name a few.

Nevertheless, fully elucidating the mechanisms governing the isomerizing cores remains an open challenge, as testified by the recent experimental discoveries regarding the non‐adiabatic thermal isomerization of azobenzene, one of the longest‐established photochromic scaffolds.^[18]^

Double‐bond isomerization is one of the key photochemical transformations studied in the field of molecular switches (Figure 1a).[^19^, ^20^] Imines (e.g., iminothioindoxyl switch, ITI, Figure 1b) are prototypical compounds that undergo such transformations. These compounds allow two archetypal motions: 1) *rotation* about the double bond of one of the substituents, which is accompanied by the movement of substituents out of the nodal plane that initially characterized the π‐bond, and 2) *inversion* around the double bond, during which one of the substituents moves in the same plane of the π‐bond.^[21]^ The nature of the electronic transition (n‐π*, see Figure 1a)^[22]^ and the concurrent presence of a lone pair on the nitrogen atom dictate that the motion in the electronically excited state proceeds via *rotation*, while in the ground state, the molecule undergoes *inversion*.

**Figure 1:**
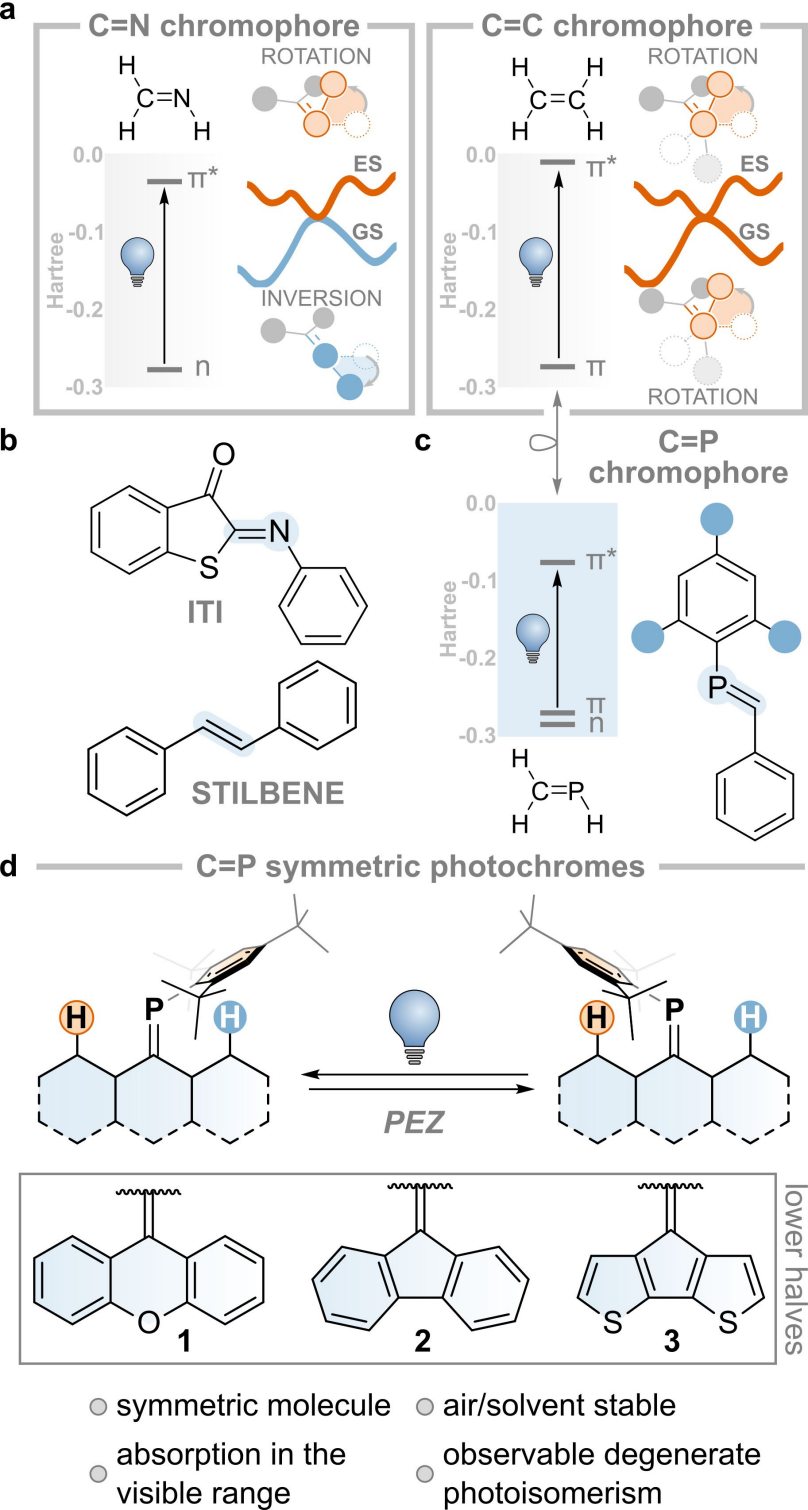
**a**. Ground and excited state isomerization modes of C=N and C=C double bonds. **b**. Examples of imine and alkene photoswitches. **c**. An example of a phosphalkene photoswitch. **d**. Present work.

By contrast, rendering the n‐orbital unavailable—either by protonation (i.e. forming an iminium) or by replacing the nitrogen for a carbon (e.g., in stilbene, Figure 1b)—forces both excited and ground state motions to happen via *rotation*, as imparted by the π and π* nature of the HOMO and LUMO (see Figure 1a).[^22^, ^23^] This knowledge allows us to interpret the difference in thermal isomerization lifetime or even to design imines as minimalistic molecular motors due to the intrinsic breaking of the symmetry of their motion in electronically excited and ground states.[^24^, ^25^, ^26^, ^27^]

In recent years, researchers have been expanding the portfolio of alkenes by exploring compounds that contain heavier main‐group elements belonging to the pnictogen group.[^22^, ^28^, ^29^]

The chemistry of such heavier pnictogen moieties is dominated by the altered π‐bonding situation involving atomic orbitals beyond the second shell. Diphosphenes, the heavier analogs of azo compounds, undergo photo‐stimulated *E*‐to‐*Z* isomerization via P=P bond rotation but revert to the thermodynamically stable *E* form under thermal conditions.[^30^, ^31^, ^32^, ^33^] Recently, the heavier distibene and dibismuthene were incorporated into photoswitchable compounds.^[34]^

The phosphorus analog of imine, known as phosphaalkene (C=P, see Figure 1c), has long been known to undergo photo‐isomerization, with the occasional possibility of isolating the metastable isomer.[^35^, ^36^] The ground state reactivity of phosphaalkenes is often associated with its isolobal congener, the C=C bond, in a comparison known as “Phosphorus: The Carbon Copy”,[^37^, ^38^] whereas the “Photocopy”, i.e. the similarities and dissimilarities of the excited state, is significantly less explored.^[39]^ Few studies have focused on understanding the mechanisms that govern the isomerization of this class of compounds, as well as on any analogy or difference with the lighter imines and alkenes. This is often related to the instability of these low valent P species, which are sensitive to oxygen and prone to undergo solvolysis. One strategy to overcome these limitations is the introduction of a bulky substituent (e.g., Mes*=2,4,6‐tri‐^*t*^butyl benzene) imparting kinetic stabilization.^[40]^

In this work, we present the synthesis of a phosphaalkene that is stable under ambient conditions (**1**, see Figure 2), the spectroscopic and computational study of its isomerization mechanism, as well those of two other phosphaalkenes previously studied by some of us (**2** and **3**).[^41^, ^42^] Their structure is based on symmetric lower halves connected to the C=P bond. Hence, they can only undergo degenerate photoisomerization, similar to what was computationally predicted for the barbiturate scaffolds^[43]^ that have inspired recent molecular motor designs.^[44]^ To the best of our knowledge, we are the first to report *experimental* evidence of degenerate photoisomerization, which we have achieved by employing photochemical‐exchange NMR spectroscopy (PC‐EXSY).^[45]^

**Figure 2:**
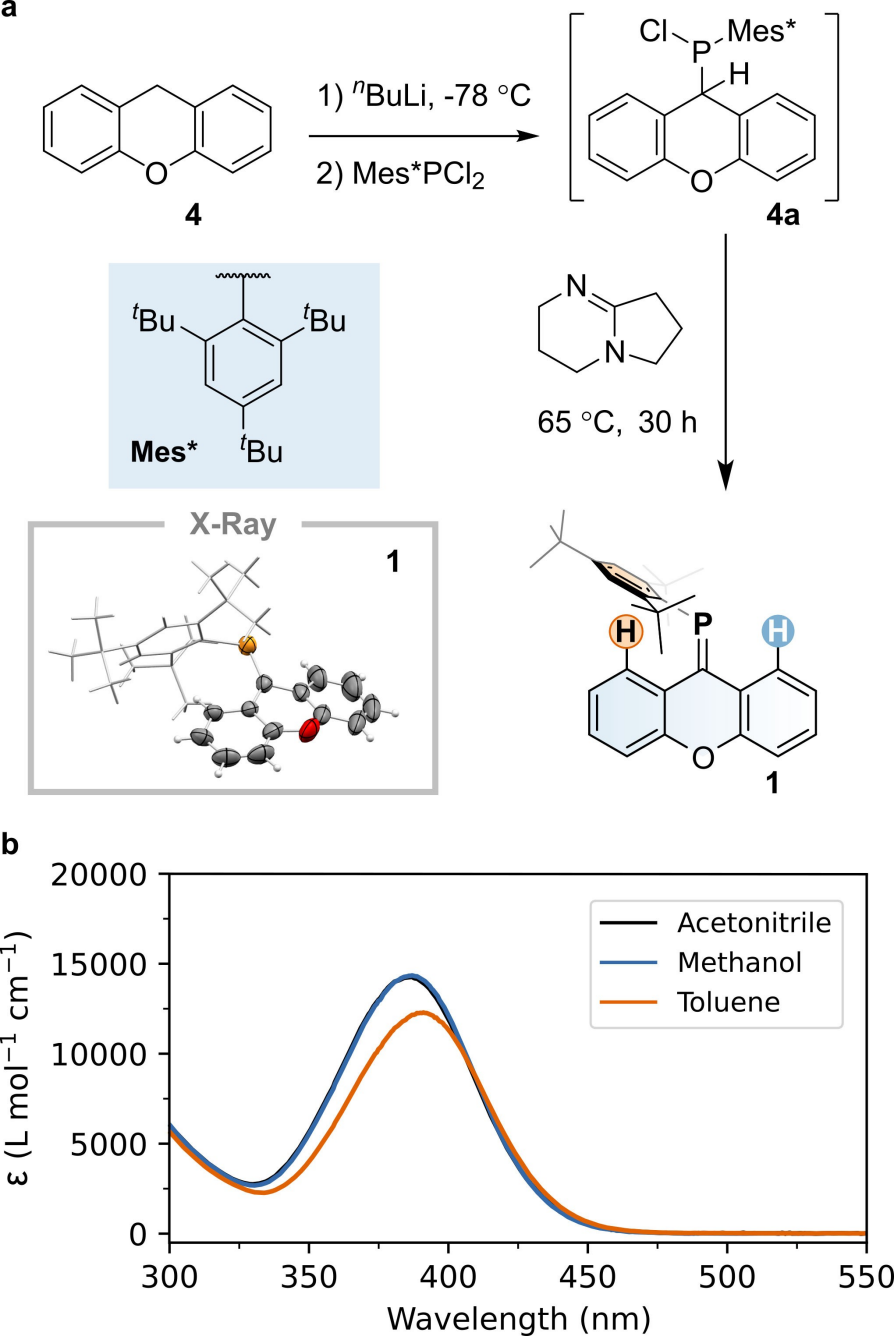
**a**. Synthesis of phosphaalkene **1**. Inset: X‐ray structure of **1**. Thermal displacement ellipsoids are set at 50 % probability levels, hydrogen atoms are omitted, and the Mes* group is shown as a wireframe for clarity. **b**. Molar absorptivity of **1** in different solvents.

To synthesize **1**, we used a single‐pot, multi‐step procedure starting from commercially available 9*H*‐xanthene (**4**, Figure 2). Treatment of **4** with ^*n*^BuLi, followed by the addition of Mes*PCl_2_, yielded chloro(9H‐xanthen‐9‐yl)(2,4,6‐tri‐^*t*^butylphenyl)phosphine **4 a**, with a characteristic ^31^P NMR resonance at 70.9 ppm (see Figure S14). Without further purification, **4 a** was treated with the non‐nucleophilic base 1,5‐diazabicyclo[4.3.0]non‐5‐ene (DBN), forming phosphaalkene **1** via dehydrochlorination, in overall 82 % yield. Compound **1** was purified by column chromatography and remains stable under ambient conditions, showing no signs of decomposition after several months. The ^31^P NMR spectrum of **1** exhibits a single sharp resonance at 219.6 ppm (see Figure S12), which is a strongly shielded signal for a normally polarized phosphaalkene (P^δ+^C^δ−^).^[46]^ Single‐crystal X‐ray diffraction studies of compound **1** reveal a P=C bond length of 1.702(4) Å, a C‐P=C bond angle of 107.6(2)° and moderate twisting of the xanthene unit (17.2(2)°). These structural parameters are consistent with those observed in related phosphaalkenes (e.g., P=C distances: 1.697/1.699 Å; C−P=C angles: 105.0/106.1°)^[40]^ and agree well with the calculated ground state model (see below). The compound is a yellow solid with a broad absorption band with molar absorptivities ranging between 10 ⋅ 10^3^ and 15 ⋅ 10^3^ M^−1^ ⋅ cm^−1^ in different solvents at ca. 400 nm with an optical onset at ca. 455 nm that is attributed to the allowed π→π* transition (see Figure 2).

Experimental verification of double‐bond isomerization in symmetric systems is challenging, as the initial and final states are chemically indistinguishable.^[43]^ A specifically developed NMR experiment, i.e. photochemical exchange spectroscopy (PC‐EXSY), allows magnetization transfer to be tracked due to a positional change of the studied nucleus.^[45]^ NMR studies conducted in a benzene‐*D*_6_ solution of **1** using a 1D‐PC‐EXSY variant allowed us to monitor magnetization transfer from the high‐ to low‐frequency aromatic resonances assigned to hydrogen atoms H8 and H1, respectively.^[45]^ Difference spectra with the selective pulse at 8.50 ppm (H8, Figure 3a) in the presence and absence of light irradiation of the sample during the mixing time (395 nm), clearly show a 1D‐correlation with the same phase at 5.95 ppm, assigned to H1. Based on this positional exchange, this experiment supports our hypothesis of the photochemically induced isomerization in this C=P symmetric system (see Supporting Information for further details). We repeated the experiment for **2** and **3**, but only compound **2** showed photochemically induced isomerism (see Supporting Information).

**Figure 3:**
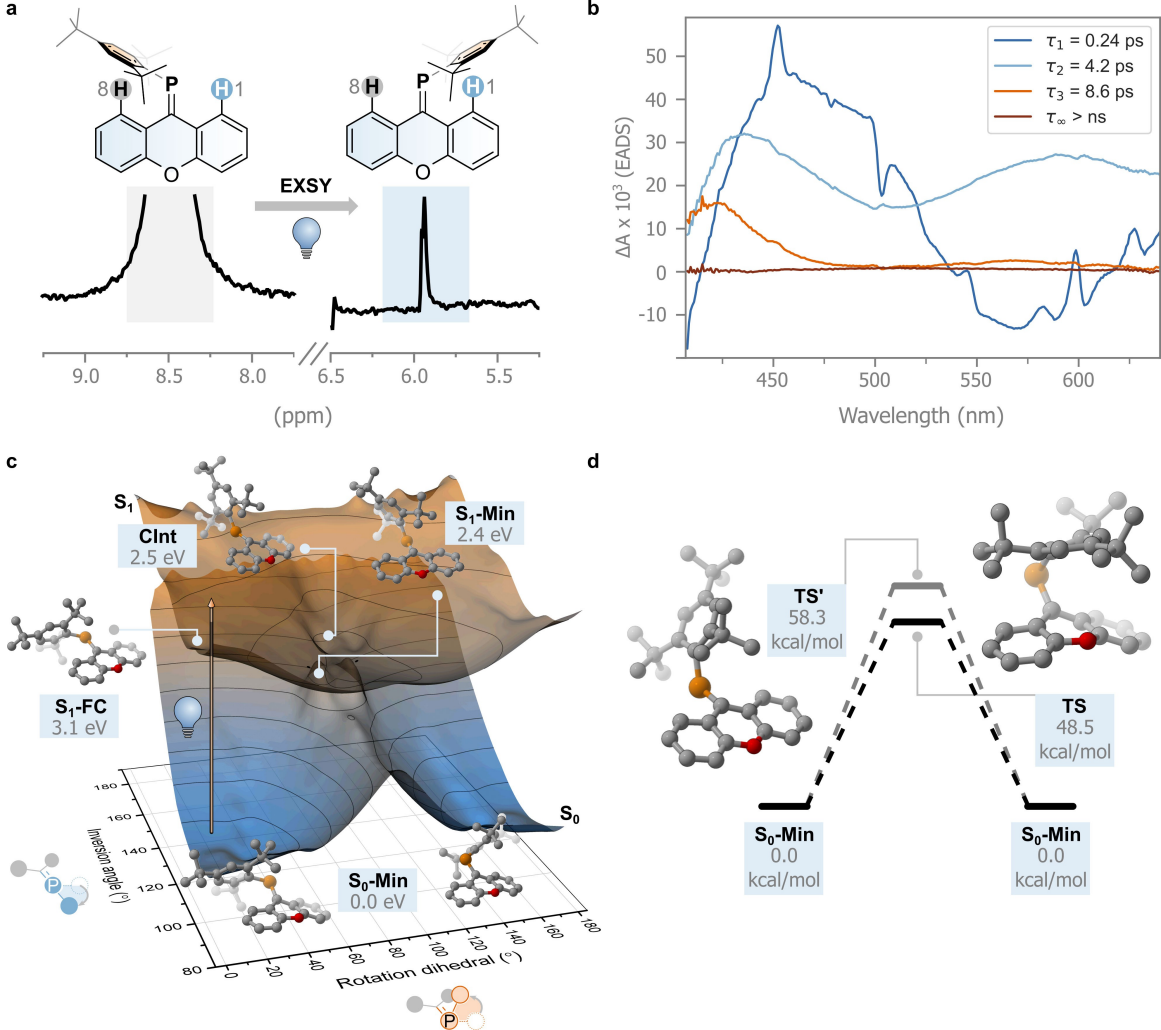
**a**. PC‐EXSY experiment. **b**. Evolution Associated Difference Spectra (EADS) obtained from a global fit of the femtosecond transient absorption spectral data recorded of **1** in toluene. **c**. Potential energy surfaces of the S_0_ and S_1_ states of **1** obtained by geometry optimization using CREST3 followed by single‐point calculation using MRSF‐TDDFT. **d**. Energy diagram of the different transition states (**TS** and **TS′**) found for the thermal double‐bond isomerization of **1** at the MRSF‐TDDFT/6‐31G*/6‐311G* level.

Further insights into the excited state behavior of **1** are obtained by studying its ultrafast transient absorption spectra with excitation at 400 nm, probing the isomerization in four solvents of different nature, toluene, acetonitrile, methanol, and glycerol i.e. aprotic, polar aprotic, protic, and protic with higher viscosity, respectively. The results, however, showed essentially the same sequence of processes regardless of the solvent (Figure 3b and Figure S1, however an additional process was observed in glycerol). Therefore, we will describe the excited state dynamics of **1** and its evolution‐associated difference spectra (EADS) in toluene (Figure 3b) and refer the reader to the Supporting Information for the remaining solvents (Figures S1 and S2).

Global analysis shows that the data can be fitted with a four‐exponential decay model. Directly after excitation, the formed transient species shows a broad excited state absorption band (ESA) at around 450 nm and a negative signal in the 500–600 nm region assigned to stimulated emission with sharper features that can be attributed to the presence of a coherent artifact. The excited state absorption band covered the bleach of the ground state. The signal evolves with a lifetime τ_1_ of 0.24 ps. At longer delays, the positive signal at 450 nm decreases in intensity, while at the same time a broad induced absorption band at 550 nm appears. This species decays with a lifetime of 4.2 ps (τ_2_), forming a longer‐lived transient with a decay time τ_3_ of 8.6 ps. During these decays, the induced absorption band at 450 nm shifts to shorter wavelengths, and both positive signals decrease in intensity, assuming a shape similar to the spectrum of the pristine **1**. Finally, the original spectrum fully recovers at longer lifetimes (>2 ns, τ_∞_).

As the ESA bands peaking at about 450 nm and 550 nm decay on a different timescale, we propose that the excited state surface gives rise to a bifurcation in the excited state decay.

The first time constant (τ_1_) is associated with the movement of the molecule from the Franck–Condon excited region. Most of the excited state population funnels to the ground state through a conical intersection within τ_2_. However, the observation of a small residual intensity peaked at 550 nm suggests that some excited state population is still present in the third spectral component and decays on a longer 8.6 ps timescale (τ_3_). Bearing in mind that an EADS does not necessarily reflect the absorption spectrum of a single species but can have contributions from different species, we hypothesize that the positive band peaked at about 420 nm observed in the third EADS reflects a ground‐state conformer with an absorption spectrum that is red shifted with respect to the main one, and that becomes populated upon decay from the excited state. In non‐viscous solvents, however, this conformer reverts back on an 8.6 ps timescale either to the initial ground state geometry or to the *degenerate* isomerized geometry, which exhibits the same UV/Vis absorption spectrum, indicated by the lack of absorbance in the EADS at long delays.

Compound **2** follows a similar evolution pathway to compound **1** (see Figure S2), but a portion of the excited‐state population decays into a long‐lived state (>ns) that we attribute to a triplet, following an excited‐state bifurcation. Compound **3** shows an even more pronounced population of a signal that we assign to a long‐lived triplet state (see Supporting Information).

This result, together with the absence of switching from our NMR data, suggests that the singlet surface could be directly involved in the isomerization process, while the triplet surface leads to other deactivation pathways.

Following these spectroscopic results, we wanted to assess whether the motion in the P=C bond is governed by the group analogy with imines or by their diagonal, isolobal, relationship with alkenes (Figure 1) on the singlet surface (Figure 3c). The P=C bond isomerization of **1** can theoretically occur via *rotation* about the P=C bond or via *inversion* of the phosphorus atom. We investigated both of these mechanisms for the electronically excited and ground states by performing a relaxed scan of the rotational dihedral and inversion angles in the ground and first excited state using the non‐self‐consistent‐field tight‐binding GFN0‐xTB level of theory (see Figure 3c for a definition of the atoms considered; see also Supporting Information).[^47^, ^48^, ^49^, ^50^] This method was proven to be successful in correctly finding minimal energy crossing points. Hence, we decided to utilize this semi‐empirical implementation following a similar strategy we used before for an imine switch.^[21]^ The energies of the obtained geometries were subsequently refined using Mixed‐Reference Spin‐Flip TD‐DFT (MRSF‐TDDFT with a 6‐31G* basis set for all atoms apart from P, for which the 6‐311G* basis set was used).[^51^, ^52^] This recently implemented level of theory can describe multiconfigurational electronic states with a fraction of the cost of typical multireference methods.

The resulting S_0_ and S_1_ potential energy surfaces are shown in Figure 3c. Gratifyingly, we find two minima in the electronically excited state as proposed by our spectroscopic model: a local minimum in the Franck–Condon region (**S_1_‐FC**, 3.1 eV) with a geometry very close to that of S_0_; and the excited state minimum (**S_1_‐Min**, 2.4 eV, rotation angle 81°) with a geometry in which the C=P bond is broken. In the former, the xanthene moiety appears to be slightly flattened compared to the ground state minimum (**S_0_‐Min**), with C=P decreasing its out‐of‐plane puckering (identified by the O−C=P angle) from 27° to 8° and increasing its bond length, from 1.69 Å to 1.81 Å. **S_1_‐Min**, on the other hand, has the Mes* group displaced perpendicularly compared to the C=P bond in its ground state minimum form. Relatively close to the geometry and energy of **S_1_‐Min**, we find a conical intersection (**CInt**, 2.5 eV, rotation angle 83°) characterized by reduced pyramidalization at the carbon atom of the C=P bond (see Figure 3c). Having reached this geometry, the molecule funnels to the ground state, proceeding towards the initial geometry or that associated with the degenerate photoisomerization product. The computations indicate that the photoinduced isomerization process proceeds via *rotation*, similar to the C=N and C=C bonds (Figure 1a). The overall efficiency of the process of degenerate isomerization is expected to be limited due to the large dimensions of the Mes* that are displaced. Indeed, the small signal observed in the 1D‐PC‐EXSY experiment hints towards a relatively inefficient process with the excited state population reverting to the initial, unchanged geometry (i.e. with Mes* near H8, see Figure 3a).

Isomerization in the ground state would not occur via the *inversion* mechanism since the energy only increases as the inversion angle goes towards 180°. Instead, we expect the isomerization to follow the *rotation* mechanism, although we find that a significant barrier of 48.5 kcal/mol is still associated with the lowest transition state **TS**. We also located another transition state, **TS′**, at higher energy (58.3 kcal/mol), which involves a concomitant *rotation* about the single bond between the phosphorus atom and the carbon atom of the Mes*‐group (see Figure 3d). This is in line with the absence of detectable thermal C=P isomerization at room temperature, as confirmed by EXSY experiments (see Figure S5, S7, S9). Hence, the C=P bond follows the isolobal analogy with the C=C bond also in its excited and ground state isomerization modes. The *inversion* mechanism in the ground state does not occur because the n‐orbital, rich in s‐character, renders the planarization of the C=P‐Mes* angle in the ground state unfeasible.

In this study, we have presented the first example of experimentally observed degenerate photoisomerization using three different phosphaalkenes **1**–**3**. Their structural differences, analyzed via ultrafast transient absorption spectroscopy and MRSF‐TDDFT calculations, elucidated the isomerization modes and demonstrated the critical connection between fundamental principles, such as the isolobal analogy between C=P and C=C bonds and the understanding of the nanoscale molecular motion. We are confident that this study will pave the way for further exploration of symmetric heteroalkene systems and expand the potential applications of main group‐based molecular machines.

## Supporting Information

The authors have cited additional references within the Supporting Information.[^54^, ^55^, ^56^, ^57^, ^58^, ^59^, ^60^, ^61^, ^62^, ^63^, ^64^, ^65^, ^66^, ^67^, ^68^, ^69^, ^70^, ^71^, ^72^, ^73^, ^74^]

## Conflict of Interests

The authors declare no conflict of interest.

## Supporting information

As a service to our authors and readers, this journal provides supporting information supplied by the authors. Such materials are peer reviewed and may be re‐organized for online delivery, but are not copy‐edited or typeset. Technical support issues arising from supporting information (other than missing files) should be addressed to the authors.

Supporting Information

## Data Availability

The data that support the findings of this study are available in the supplementary material of this article.
