# Supplementary material for: Probing the Hidden Photoisomerization of a Symmetric Phosphaalkene Switch
Source: Angew Chem Int Ed Engl. 2024 Dec 13;64(5):e202419943. doi: 10.1002/anie.202419943 (PMC11773101; doi:10.1002/anie.202419943)
Supplement: Supplementary file 1 — Supporting Information [file ANIE-64-e202419943-s001.pdf]

Angewandte Chemie
Eine Zeitschrift der Gesellschaft Deutscher Chemiker

Supporting Information

Probing the Hidden Photoisomerization of a Symmetric Phosphaalkene Switch

R. Deka, J. D. Steen, M. F. Hilbers, W. G. Roeterdink, A. Iagatti, R. Xiong, W. J. Buma, M. Di Donato, A. Orthaber, S. Crespi**

Table of Contents

1. Experimental Procedures 2

1.1. General details 2

1.2. X-ray Crystallographic Details 2

1.3. Reagent Information 3

1.4. Transient Absorption Spectroscopy 3

1.5. Computational Analysis 7

1.6. 1D-PC EXSY studies 9

1.7. Synthetic procedure 14

2. Mechanistic Interpretation 18

3. References 19

1. Experimental Procedures

1.1. General details

All manipulations were performed under an atmosphere of purified argon using the standard high-vacuum Schlenk-line technique. Argon was supplied by Air Liquide Europe. All glassware was flame-dried and cooled under vacuum, and purged with argon before use. Solvents were freshly collected from *Pure Solve MD7* solvent purification system under argon. NMR spectra were recorded on a Jeol Eclipse+ 400 (^{1}H, 400 MHz; ^{13}C, 101 MHz; ^{31}P, 162 MHz) spectrometer at 298 K unless noted otherwise. Chemical shift values are quoted in δ (ppm) and coupling constants in *J* (Hz). ^{1}H chemical shift values are reported relative to tetramethylsilane (TMS) and referenced to the residual proton resonances of the corresponding deuterated solvent signal. ^{13}C{^{1}H} NMR spectra are reported relative to TMS using the natural-abundance carbon resonances of the deuterated solvents. ^{31}P NMR spectra are referenced externally to H_3PO_4 (85%, aq.). The following abbreviations (or combinations thereof) were used to describe multiplicities: s, singlet; d, doublet; t, triplet; m, multiplet, brs, broad singlet. High-resolution mass spectra (HR-MS) were recorded on a Thermo Scientific Orbitrap LTQ XL spectrometer.

1.2. X-ray Crystallographic Details

Single crystals were mounted on a fiber loop and fixated using Fomblin oil. The data were collected on a Bruker D8 APEX-II equipped with an APEX-II CCD camera using MoKα radiation (λ = 0.71073 Å). Data reduction was performed with SAINT, absorption corrections for the area detector were performed using SADABS.[1] Structures were solved by direct methods and refined by least squares methods on F^2 using the SHELX and the OLEX2 software suites, respectively.[2,3] All the non-hydrogen atoms were refined using an anisotropic model and all the hydrogen atoms were constrained in geometrical positions to their parent atom. Crystallographic data are presented in Table S1. Deposition number CCDC 2387089 contains the supplementary crystallographic data for this paper. These data can be obtained free of charge via www.ccdc.cam.ac.uk/data_request/cif, or by emailing data_request@ccdc.cam.ac.uk, or by contacting The Cambridge Crystallographic Data Centre, 12 Union Road, Cambridge CB21EZ, UK; fax:+441223336033.

1.3. Reagent Information

^{n}BuLi (1.6 M in hexane), 9*H*-xanthene, and 1,5-diazabicyclo(4.3.0)non-5-ene (DBN) were purchased from Sigma-Aldrich and used as received. For column chromatography, silica gel (40-63 μm) from VWR was used. A gradient elution using pentane and toluene was performed, utilising Merck aluminium TLC sheets (silica gel 60F254). All solvents were purchased from VWR. Dichloro(2,4,6-tri-tert-butylphenyl)phosphane (Mes*PCl_2) was prepared according to literature methods.[4] Compounds **2** and **3** were synthesized according to literature procedures.[5,6] The reader is referred to the original publications for additional information on the synthesis, characterization, and optical properties of these compounds.

1.4. Transient Absorption Spectroscopy

Time-resolved UV-Vis spectroscopy for compound **1** in MeCN, toluene and MeOH was performed using the output of a regeneratively amplified Ti:sapphire laser (Spectra Physics Solstice Ace). 800 nm 100 fs pulses were used to generate the pump light at 400 nm using an optical parametric amplifier (Spectra Physics OPA-C), and the probe light using supercontinuum generation. The pump and probe beams were overlapped onto a 2 mm quartz flow cell holder. Before generating the probe, the beam was sent through a delay stage, allowing time control between the pump and probe beams at the sample. The polarization between the pump and probe beams was set at the magic angle to avoid anisotropic effects. The probe beam was then passed through the sample with the residual 800 nm filtered using a short pass filter, and the light was directed onto the slit of a spectrograph (Andor Kymera 193i), focused onto a grating and dispersed onto a 512 pixel CCD camera. Half of the pump pulses were blocked using a synchronized chopper, allowing the measurement of the intensity of the probe with and without the pump present on the sample. The difference absorbance was then calculated as:

$$\Delta Abs = -\,{}^{10}\log\frac{I_{pump+probe}}{I_{onlyprobe}}$$

The measured energy at the sample was 1.0 mJ. We used a sample with an optical density of about 0.5 OD. The sample was mounted on an automated, movable, two-axis stage, controlled by in-house software to avoid bleaching of the solution. The data was processed with Glotaran 1.5.1[7] using global analysis, applying a linear decay kinetic scheme with four time constants, of which one was taken as infinite on the time scale of the experiments.

The apparatus used for the transient absorption spectroscopy measurements of compounds **1** (in glycerol), **2** and **3** (in toluene) has the following specifications: 80 fs pulses centered at 810 nm were produced by an integrated home-made Ti:sapphire oscillator coupled with a

regenerative amplifier system (Amplitude Pulsar). The excitation wavelength was set at 400 nm, obtained as the second harmonic of the fundamental laser radiation, and excitation power was set at 30-50 nJ for all measurements. The pump beam polarization has been set to magic angle with respect to the probe beam by rotating a $\lambda\backslash 2$ plate, to exclude rotational contributions. The white light probe pulse was generated by focusing a small portion of the fundamental laser radiation on a 3 mm thick CaF_2 window. A portion of the generated white light was sent to the sample through a different path and used as a reference signal. After passing through the sample the white light probe and reference pulses were both directed to a flat field monochromator coupled to a home-made CCD detector. Transient signals were acquired in a time interval spanning up to 500 ps. The sample was contained in a 2 mm quartz cuvette, mounted on a movable holder in order to minimize photodegradation. Measurements were performed at room temperature. Concentrations were adjusted to an absorbance of 0.9 – 1.0 OD (for the respective optical path) at the absorption maximum, which amounted to about 0.3 – 0.5 OD at the excitation wavelength. Before and after the measurements, the integrity of the sample was checked on a PerkinElmer LAMBDA 950 spectrophotometer. The data was analyzed by means of singular value decomposition, global and transient analysis, employing the software Glotaran 1.5.1.[7]

The kinetic heatmaps and the EADS for compound **1** in acetonitrile, methanol and toluene are reported in Figure S1. In these cases, we observed a recovery of the original signal at long delay times (> ns). It is worth noting that due to the limitations of the camera used and, more importantly, the presence of the laser signal that covers the band associated with ground state bleach recovery, we cannot completely rule out the presence of long-lived components (e.g., triplet states) for these cases. Nevertheless, the contributions from these species appear to be either nonexistent or marginal. For compound **1**, to properly fit the data (mostly due to the noise generated by the laser at ca. 400 nm), the model with four components was better than the one with three components. In particular, the long kinetic component in non-viscous solvents (close to 0 ΔOD) allowed us to conclude that the initial population has recovered during the delay times of our setup.

On the other hand, when **1** is dissolved in glycerol we can observe a significant change in the excited state kinetics. The fastest EADS now lacks the stimulated emission component observed in less viscous solvents, while the motion on the excited state surface is considerably slowed (e.g., τ_1=0.17 vs 0.9 ps for the lifetime of the first component in MeOH vs. glycerol, see Figures S1 and S2). The evolution of the excited state motion proceeds with a shift of the broad absorption band peaking at ca. 625 nm to the blue region of the spectrum (ca. 575 nm,

τ_2=14 ps) and finally to a component peaking at 430 nm (τ_3=252.3 ps) associated with the population of a distorted conformer. Indeed, the band peaking at about 430 nm persists at long delay times (τ_4=100 ns). This component has characteristics dissimilar from a trace typical for excited states (i.e. broad absorption in the red region of the spectrum) and it is too long-lived to be a thermalization.

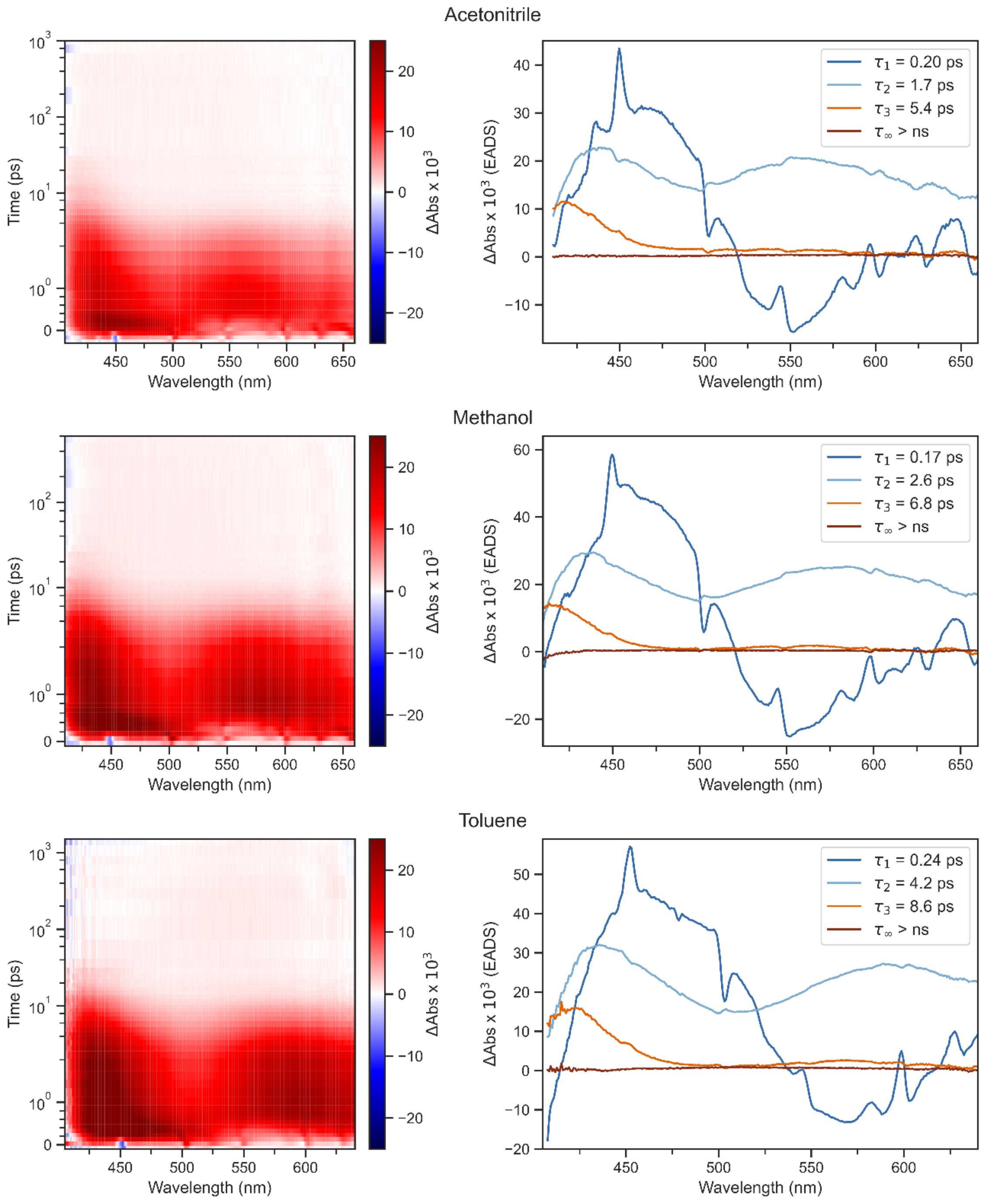

Figure S1. Kinetic Heatmaps after excitation and the Evolution Associated Spectra (EADS) obtained from a global fit of the femtosecond transient absorption spectral data recorded of **1** in (top) acetonitrile, (middle) methanol, and (bottom) toluene. Laser pump is at 400 nm.

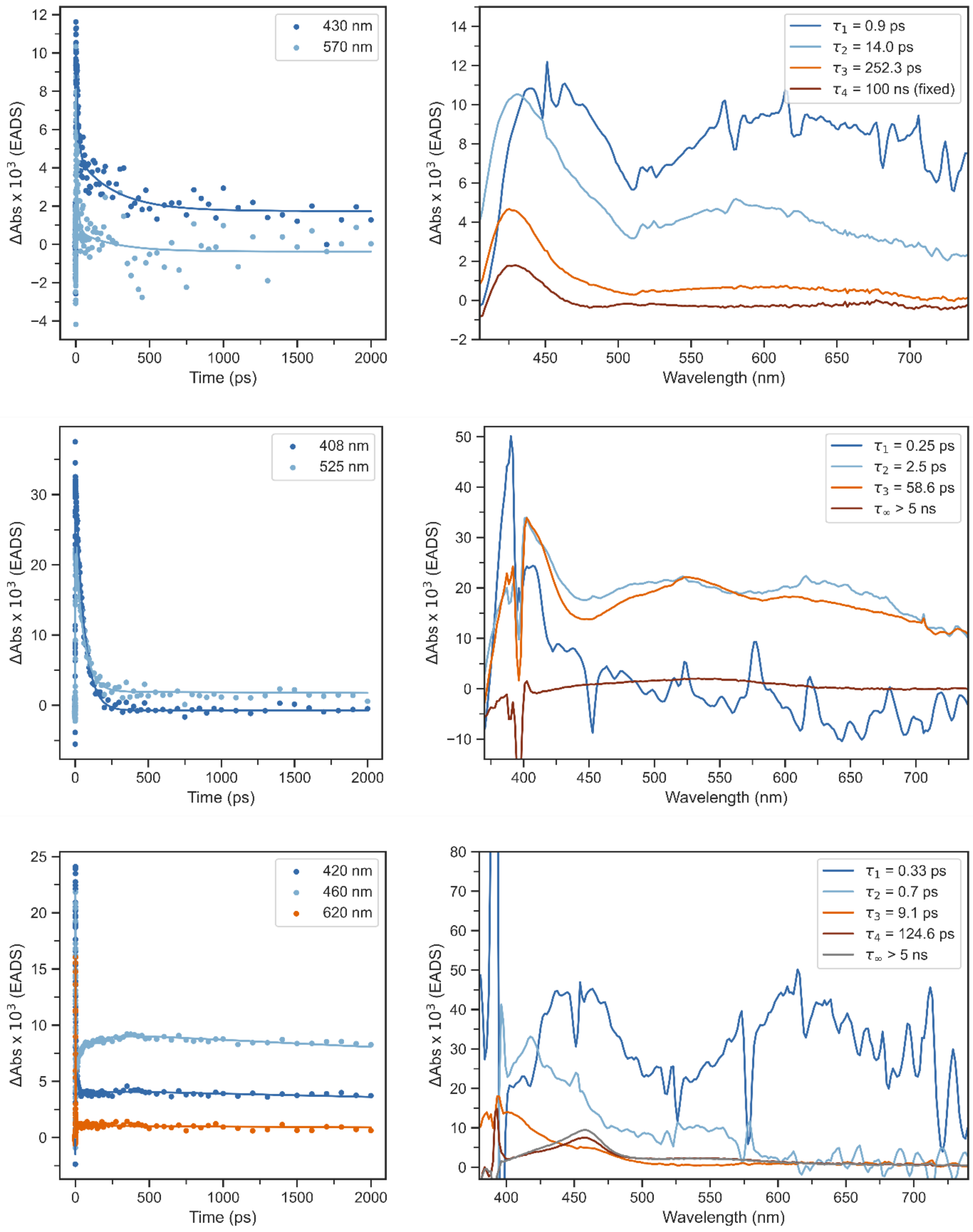

Figure S2. Kinetics and Evolution Associated Spectra (EADS) obtained from a global fit of the femtosecond transient absorption spectral data recorded of (top) **1** in glycerol, (middle) **2** in toluene, and (bottom) **3** in toluene. Laser pump is at 400 nm.

Compound **2** in toluene has a different excited state evolution. As for compound **1**, it possesses extremely fast dynamics immediately after the laser pulse (τ_1=0.25 ps), associated with a

relaxation out of the Franck-Condon minimum, possibly induced by a fast solvent response. No stimulated emission was observed in this case. The excited state further relaxes within τ_2=2.5 ps, and an absorption appears at around 550 nm, possibly indicating the motion far from the original geometry populated upon the laser pulse absorption. The excited state decays within τ_3=58.6 ps, and a long-lived species (τ_∞ > 5 ns) develops with broad absorption centered at 525 nm. The lack of full recovery of the ground state bleach, observable at ca. 400 nm, allowed us to assign the broad non-decaying band to a long-lived triplet state, which originated from an excited state bifurcation. The majority of the excited state population still funnels through the conical intersection towards the ground state, populating either the initial ground state geometry or the *degenerate* isomerized geometry, while the remaining part of the population deactivates through intersystem crossing, populating the long-living triplet.

Finally, compound **3** presents a third case in our set of examples. Its overall initial evolution is relatively similar to **1** and **2**, with a broad absorbing excited state that covers the entire range of our camera with maxima at ca. 450 and 625 nm. Ultrafast relaxation (τ_1=0.33 ps) is followed by a very rapid excited state decay (τ_2=0.7 ps), which mostly populates the distorted conformer absorbing at ca. 400 nm. Similar to compound **2**, in this case, a long-living state is populated in about 9.1 ps, presenting an absorption band that peaks at ca. 460 nm, possibly representing a triplet state. The intensity of the 460 nm band slightly increases within 124.6 ps, possibly signaling a structural relaxation occurring on the triplet surface. The intensity of the final component remains constant within the probed time range.

1.5. Computational Analysis

The 3.0 pre-release development fork of CREST[8] was used to optimize the constrained geometries for the scan of the rotation dihedral angle and inversion angle at the non-self-consistent GFN0-xTB level (Figure S3), both for the S_0 and the S_1 state.[9–11] Subsequent single-point calculations were performed using Mixed-Reference Spin-Flip TD-DFT (MRSF-TDDFT)[12,13] in GAMESS[14] (version 30 Sep 2023 R2) at the BHHLYP[15] level with the following basis sets: for H, Pople's 6-31G split valence basis set[16]; for C and O, Pople's 6-31G split valence basis set[17] with polarization[18] (i.e. 6-31G*); for P, McLean-Chandler basis set[19] including an extra polarization function[20] (i.e. 6-311G*). All single point calculations at this theory level described below were conducted using the aforementioned basis sets. Because of the symmetry of **1**, only the geometries with rotation dihedral angles between 0-90° were calculated. Subsequent mirroring of the calculated energies along this coordinate afforded the potential energy surfaces over the whole 0-180° range.

The transition states (**TS** and **TS'**) were first found using pysisyphus[21] at the GFN2-xTB level and were reoptimized using ORCA 5.0.4[22,23] at the r^{2}SCAN-3c level[24] with broken symmetry.

The single-point calculation for **TS** was subsequently performed using MRSF-TDDFT in GAMESS (*see above*). However, this calculation for **TS'** yielded unrealistically high relative energies (95.8 kcal/mol). We, therefore, resorted to obtaining the single-point energy in an alternative way: 1) generate geometries (*images*) between **TS** (which showed a reasonable relative energy of 48.5 kcal/mol) and **TS'** using NEB-IDPP[25,26] with ORCA; 2) calculate energies using MRSF-TDDFT in GAMESS (*see above*) of each *image* sequentially (from **TS** to **TS'**), each time using the orbitals from the previous single-point calculation as guess orbital. In this way we obtained a more reasonable energy of 58.3 kcal/mol *vs* **S_0-Min**.

Geometry optimization of the minima at the ground (**S_0-Min**) and excited state (**S_1-FC** and **S_1-Min**) was performed with MRSF-TDDFT in GAMESS (*see above*).

A guess geometry for the conical intersection between S_0 and S_1 (**CInt**) was found first using the 3.0 pre-release of CREST (*see above*), which was subsequently optimised using ORCA 5.0.4[22,23] spin-flip TDDFT (SF-TDDFT)[27] at the BHHLYP[15] level with the 6-31G(d) basis set.[17,20] The single-point energy was subsequently calculated using MRSF-TDDFT in GAMESS (*see above*).

The cartesian coordinates of the optimized structures are provided as .xyz files (including the energy as a comment on the second line) in the figshare repository with the following DOI: 10.6084/m9.figshare.27088969.

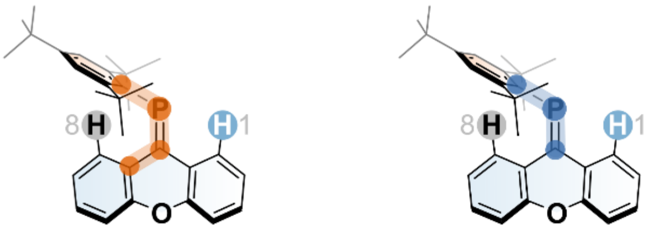

Figure S3. The rotation dihedral angle (orange) and inversion angle (blue) of **1** used for optimization of the constrained geometries.

1.6. 1D-PC EXSY studies

A slightly adapted pulse sequence was implemented for a Bruker Avance neo 500 MHz Basic NMR Spectrometer operated by TopSpin version 4.1.4. The samples are measured following the selective 1D version of the PC-EXSY sequence reported in Figure S4a. The more interested reader should refer to Stadler et al., ref. [45] in the main text. In this reference, it is possible to also retrieve the original pulse code for the selective PC-EXSY experiment for Bruker machines. The pulse is a modification of the SELNOGP Bruker sequence. It consists of the recycling delay D1, five radio-frequency (RF) pulses, and the acquisition time during which the signal is recorded. The first RF pulse is a 90° pulse, followed by a selective pulse (180° shaped pulse with flanking gradients) followed by a 90° pulse. During the mixing time a square 180°pulse is flanked by two opposite gradients, and ultimately sampled with a 90° pulse recording the FID. This pulse sequence represents a standard selective NOE or EXSY experiment with gradient enhancement. In addition to the original SELNOGP pulse, light from a fiber immersed in the NMR tube is switched on during the mixing time and off during the remainder of the experiment, controlled via analog output of the console (OUT2). For the experiments, we have chosen the following recycling delay and mixing time: D1=7s and D8=1.5 s. These values are in accordance with the original publication of Stadler et al. We have also used longer mixing and delay time for compound **1** to explore the range of suitable mixing times, identifying 1.5 s to be an ideal compromise for the improved intensity of the PC-EXSY signal. A pictorial representation of the output of the measure is presented in Figure S4b.

In situ illumination was performed with a 365 nm LED from Thorlabs (M365FP1, mounted to a 50 mm long heat sink, fiber coupled with SMA connection, powered by 1.2 A) using a Thorlabs M59L glass fiber with a 1000 μm core diameter, 0.50 NA and SMA connectors. One end of the fiber was cut, stripped from all coating and cladding, and sanded manually with sandpaper at the end (approx. 5 cm, see Figure S4c). The fiber is covered and protected by a 3 mm NMR tube, which is fixed in place by its cap, previously drilled to insert the unstripped part of the fiber. The drilled NMR tube cap (and consequently the 3mm NMR tube itself) is fixed in place with heat shrink tubing (the black covering in Figure S4c). An additional NMR cap (for 5 mm tubes) is drilled and inserted on the internal 3 mm tube. This cap allows the fixing of a 5 mm NMR tube, which contains the solution of **1-3** in benzene-d6, to be irradiated (Figure S4c, right). The communication between the NMR and the LED was performed via a Thorlabs LEDD1B driver, set in trigger mode, via a BNC cable connected to the OUT2 exit of the NMR control panel (Figure S4d).

The spectra are processed with standard Fourier transformation, applying slightly larger exponential apodization function (2-2.5 Hz) than standard and phased with the selective irradiation peak in the positive direction.

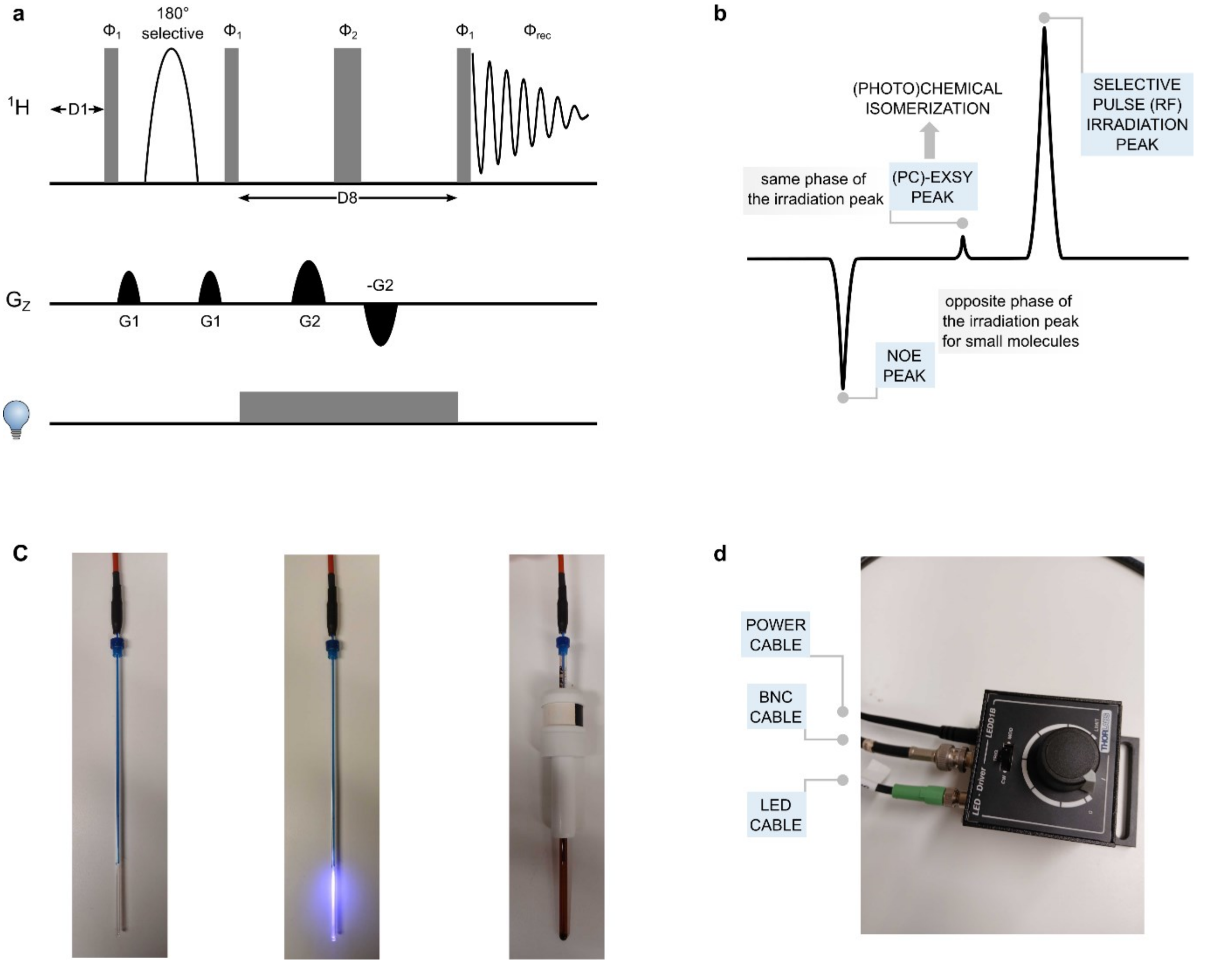

Figure S4. a. The modified SELNOGP pulse sequence used for the 1D-PC-EXSY experiment. D1=recycling delay; D8=mixing time; Φ_1=90° pulse; Φ_2=180° pulse. Phase cycling: Φ_1= Φ_2 = Φ_{rec} = x. Gradients (G_Z): G1 (square, 1ms)=15%; G2 (square, 1ms)=40%. Light channel: the square represents light on. **b.** Pictorial representation of the interpretation of the 1D-PC-EXSY experiment. It is important to underline that EXSY and PC-EXSY peaks can be observed on the same peak. **c.** Left: stripped and sanded fiber protected by a 3mm NMR tube, with a 5 mm NMR tube cap inserted; Center: light switched on; Right: fiber inserted into a 5 mm NMR tube with sample and spinner. **d**. Driver of the LED set in trigger mode.

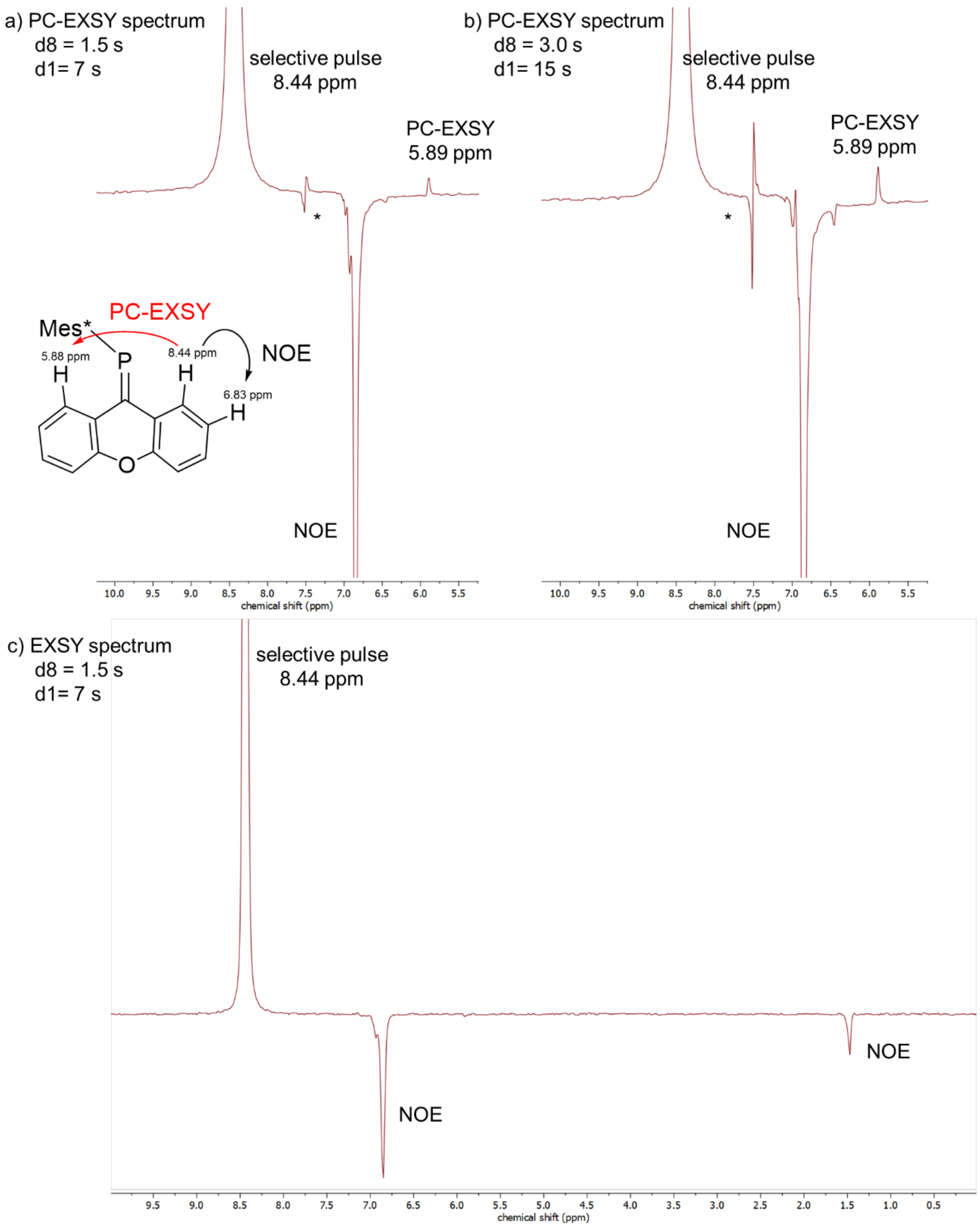

Figure S5. 1D-PC-EXSY spectrum of compound **1** showing the in-phase PC-EXSY and opposite-phase residual NOE correlations. a) mixing time = 1.5 s and delay time = 7 s. b) mixing time = 3 s and delay time = 15 s. c) Standard EXSY experiment with mixing time = 1.5 s and delay time = 7 s. * indicates dispersive artifacts from field instability and unequal perturbation resulting in selective population transfer.

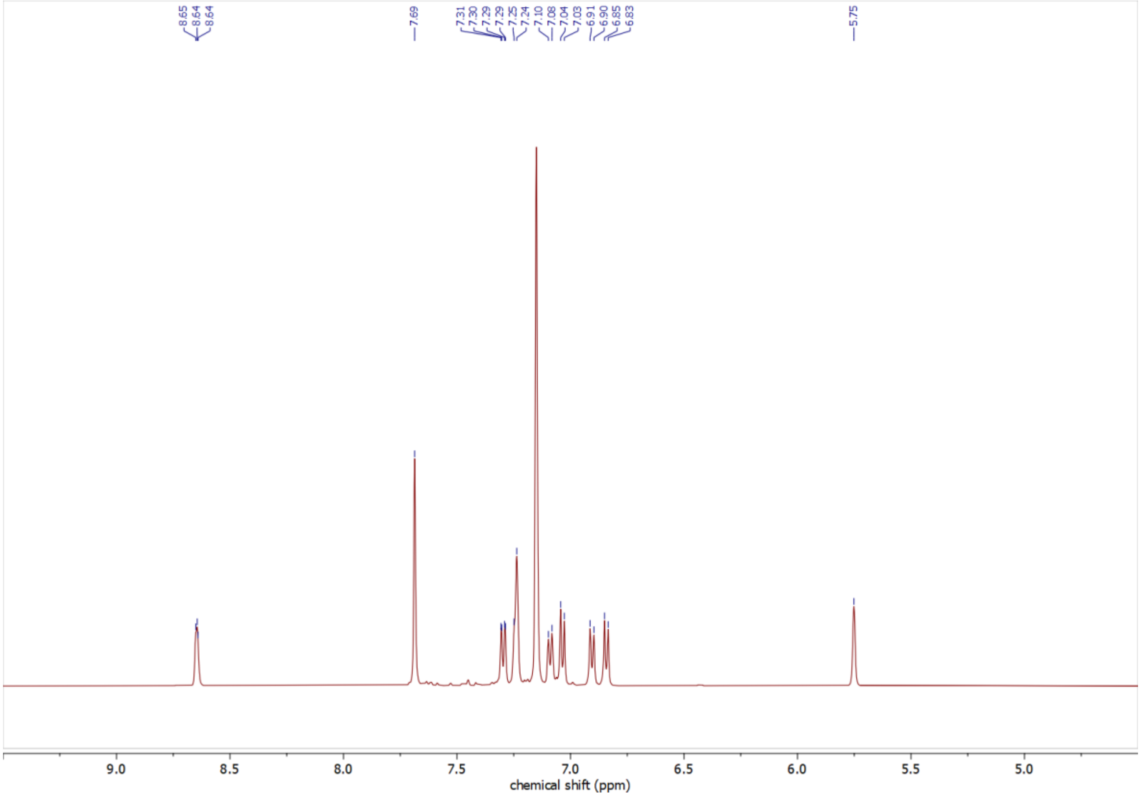

Figure S6. ^{1}H NMR spectrum of compound **2** (selected aromatic region) in benzene-d$_6$.

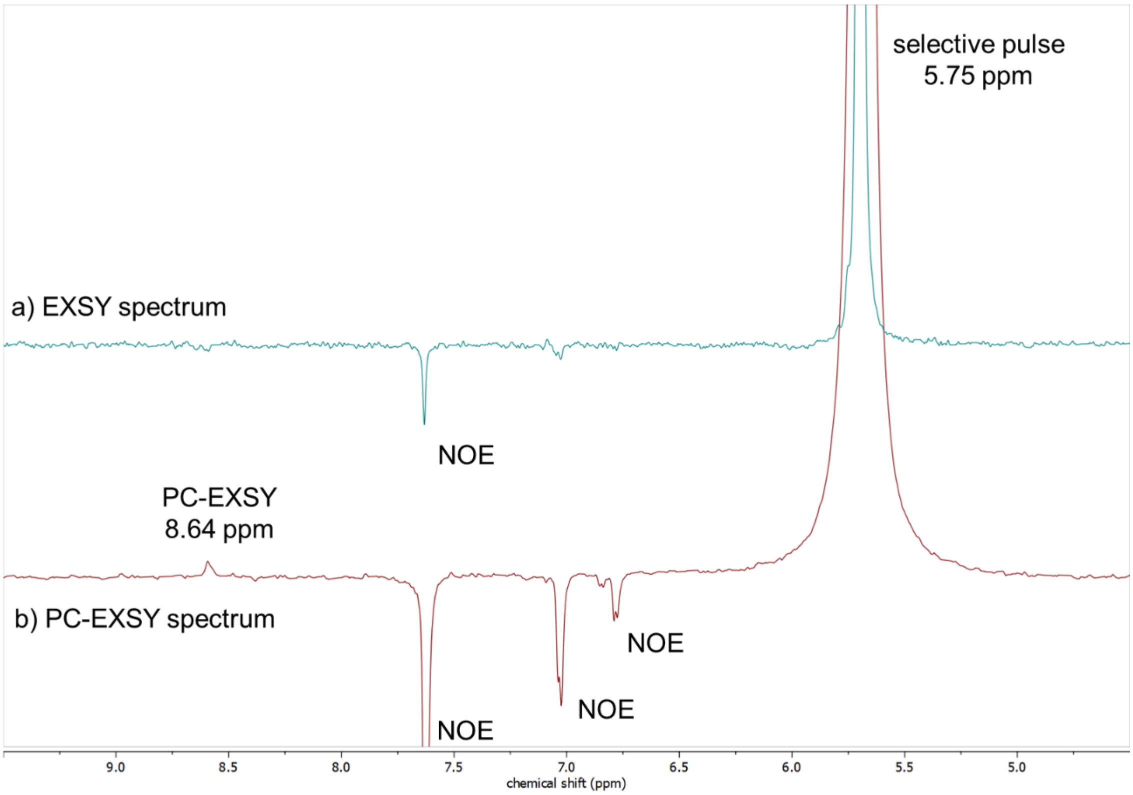

Figure S7. a) Standard EXSY and b) PC- EXSY spectra of compound **2** (selected aromatic region) in benzene-d$_6$. Mixing time 1.5 s and delay time 7 s.

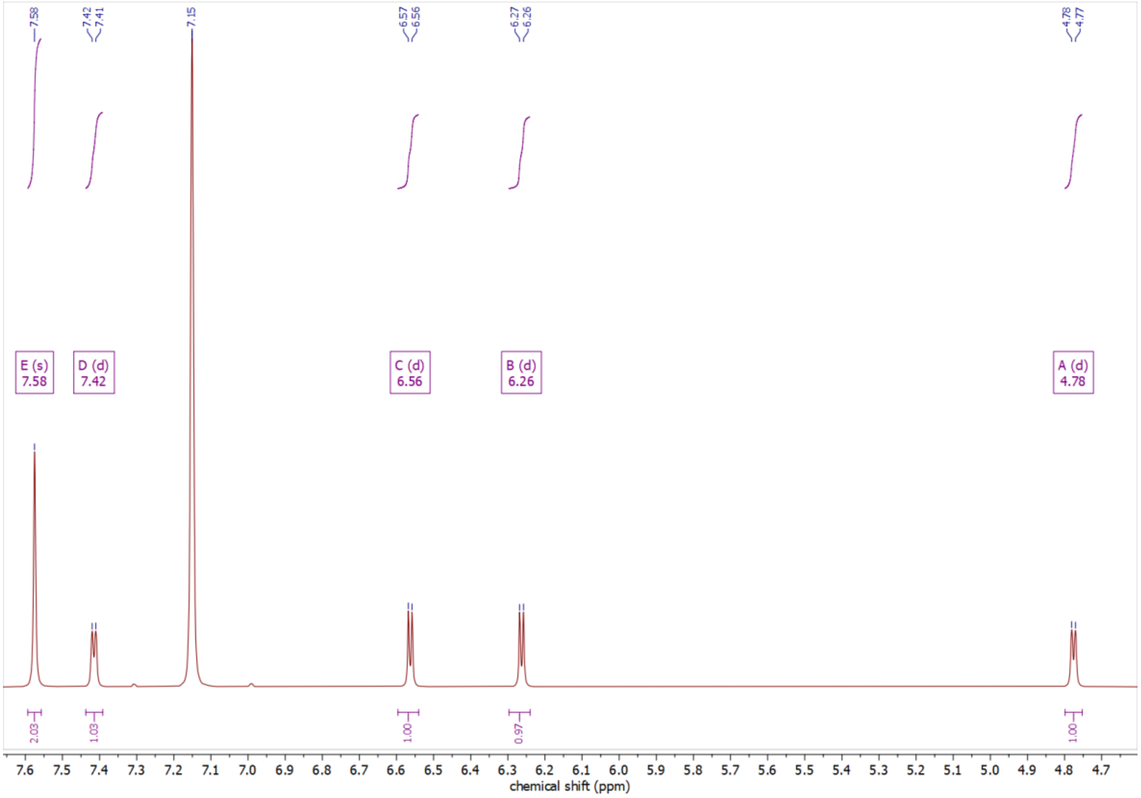

Figure S8. ^{1}H NMR spectrum of compound **3** (selected aromatic region) in benzene-d_6.

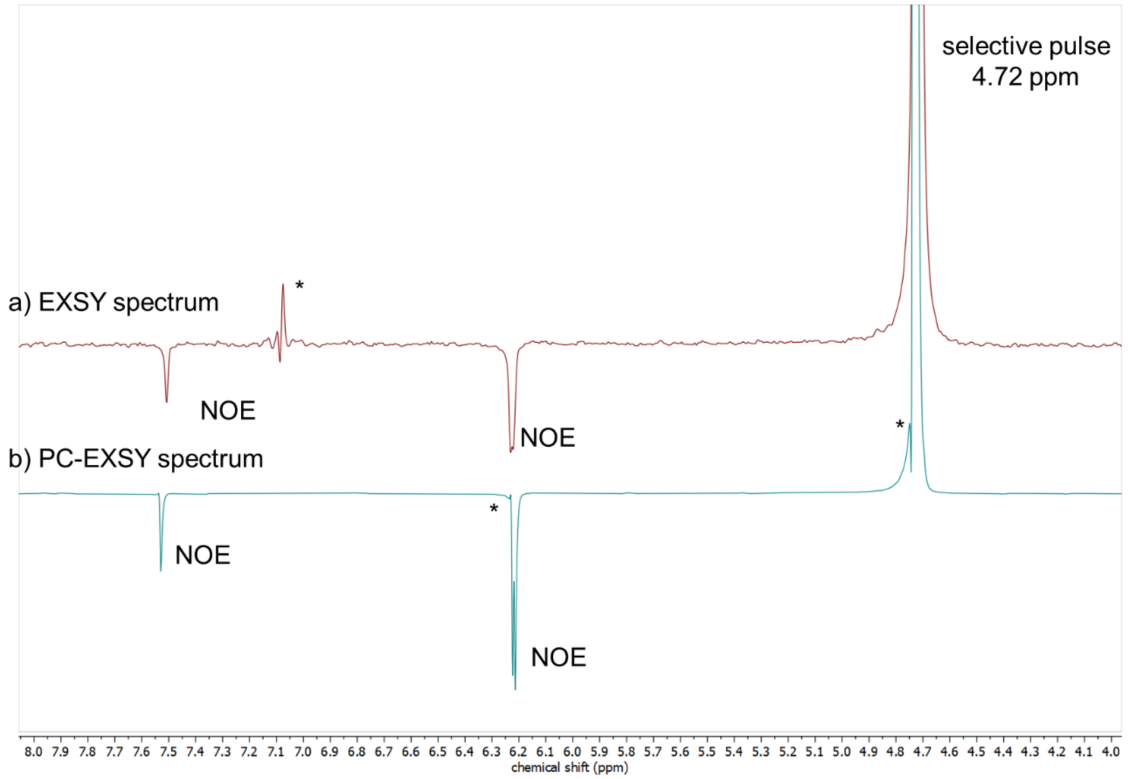

Figure S9. a) Standard EXSY and b) PC- EXSY spectra of compound **3** (selected aromatic region) in benzene-d_6. Mixing time 1.5 s and delay time 7 s. * indicates dispersive artifacts from field instability and unequal perturbation resulting in selective population transfer.

1.7. Synthetic procedure

9*H*-xanthene, **4** (0.20 g, 1.10 mmol) was dissolved in 30 mL of anhydrous THF in a Schlenk flask. The resulting solution was cooled to −78 °C and 1.6 M ^{n}BuLi (0.72 ml, 1.15 mmol) was added dropwise. The reaction mixture was stirred at −78 °C for 2 hours. Mes*PCl_2 (0.40 g, 1.15 mmol) was added to the reaction mixture, and the temperature was gradually brought up to room temperature and stirred overnight. The reaction mixture containing chloro(9H-xanthen-9-yl)(2,4,6-tri-tert-butylphenyl)phosphine (**4a**) was treated with DBN (0.15 mL, 1.15 mmol) and was stirred at 65 °C for 30h. The solvent was removed in vacuo to get a yellow solid which was purified by column chromatography using '4% toluene-96% pentane' solvent combination as eluent to obtain the desired phosphaalkene **1**.

4a: ^{31}P NMR (162 MHz, reaction aliquot in THF) δ= 70.9 ppm

1: Yellow solid; Yield: 0.41 g (82%); ^{1}H NMR (400 MHz, Benzene-D_6) δ 8.44 (ddd, J = 7.9, 5.3, 1.6 Hz, 1H), 7.51 (d, J = 1.2 Hz, 2H), 7.02 – 6.89 (m, 3H), 6.85 (ddd, J = 8.5, 7.1, 1.5 Hz, 1H), 6.75 (ddt, J = 8.4, 7.1, 1.7 Hz, 1H), 6.45 (ddd, J = 8.4, 7.1, 1.4 Hz, 1H), 5.89 (dt, J = 8.2, 1.9 Hz, 1H), 1.47 (s, 18H), 1.31 (s, 9H) ppm;^{13}C NMR (101 MHz, Benzene-D_6) δ 162.5 (d, J = 49.9 Hz), 155.4, 151.1, 149.4 (dd, J = 12.2, 4.8 Hz), 135.8 (d, J = 59.4 Hz), 129.6 (d, J = 5.4 Hz), 129.0 (d, J = 4.1 Hz), 128.4 (d, J = 5.2 Hz), 128.3, 126.6, 126.4, 125.0, 124.6, 124.0, 122.5, 116.6, 38.1, 34.9, 32.4 (d, J = 7.0 Hz), 31.4 ppm; ^{31}P NMR (162 MHz, Benzene-D_6) δ 219.6 ppm; HR-MS (APCI, Toluene) m/z: calcd for $[M]^+$ ($C_{31}H_{37}OP$) 456.2576; found: 456.2576.

Table S1. Refinement details for the X-ray structure of **1**.

Empirical formula	$C_{31}H_{37}OP$	Z	4
Formula weight	456.57	ρ_{calc}g/cm^3	1.114
Crystal System	Orthorhombic	μ/mm^{-1}	0.121
Space group	*Pna2₁*	GOF	1.043
T/K	296.15	*2ϑ* range (deg)	4.59-53.49
a [Å]	16.365(2)	Refs collected	23421
b [Å]	9.3749(14)	Final *R* indexes [*I>=2σ(I)*]	R_1 = 0.0550, wR_2 = 0.1204
c [Å]	17.747(3)	Final *R* indexes [*all data*]	R_1 = 0.0889, wR_2 = 0.1356
α [°]	90	Data / restraints / parameters	5793/46/338
β [°]	90	Independent reflections	5793 [R_{int}=0.0435, R_{sigma}=0.0429]
γ [°]	90	Radiation	MoKα (λ=0.71073)
V [A^3]	2722.7(7)	Flack parameter	0.51(4)

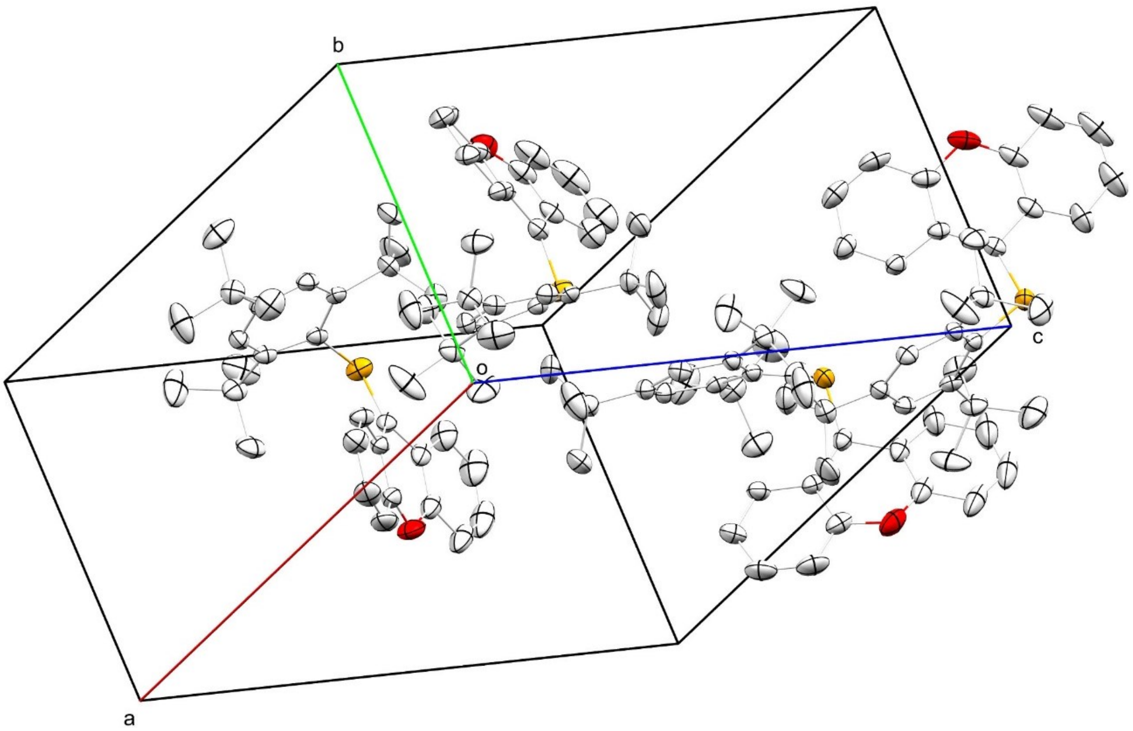

Figure S10. Packing diagram for **1**.

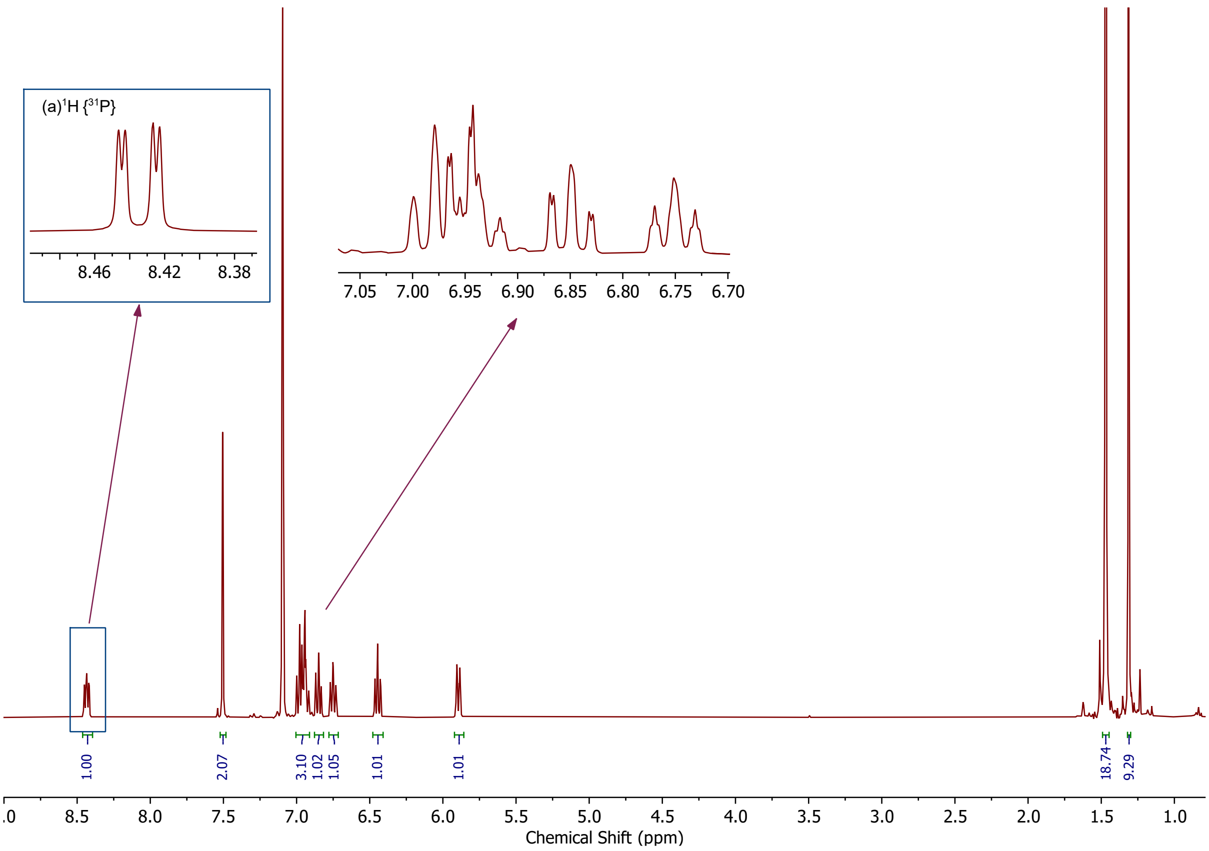

Figure S11. ^{1}H NMR spectrum of compound **1**. Inset showing (a) $^{1}H\{^{31}P\}$.

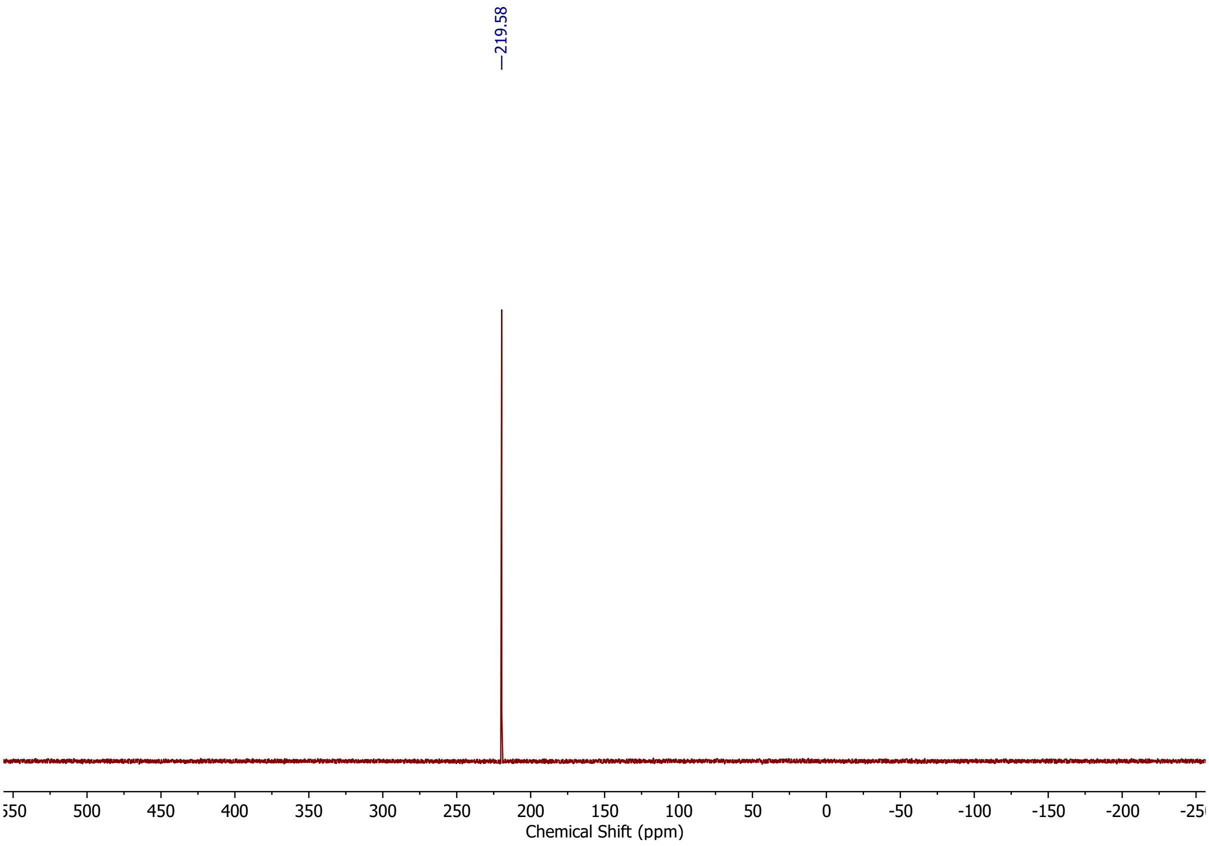

Figure S12. ^{31}P NMR spectrum of compound **1**.

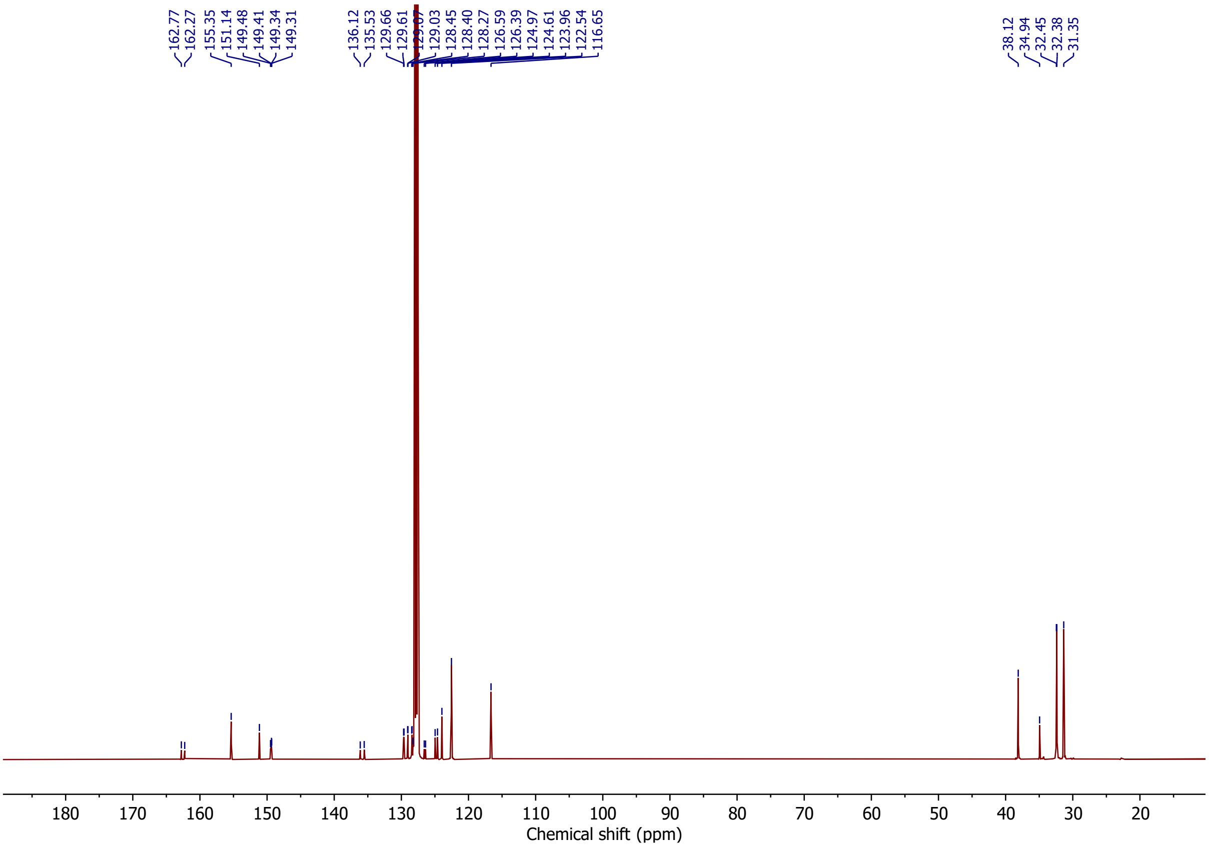

Figure S13. ^{13}C NMR spectrum of compound **1**.

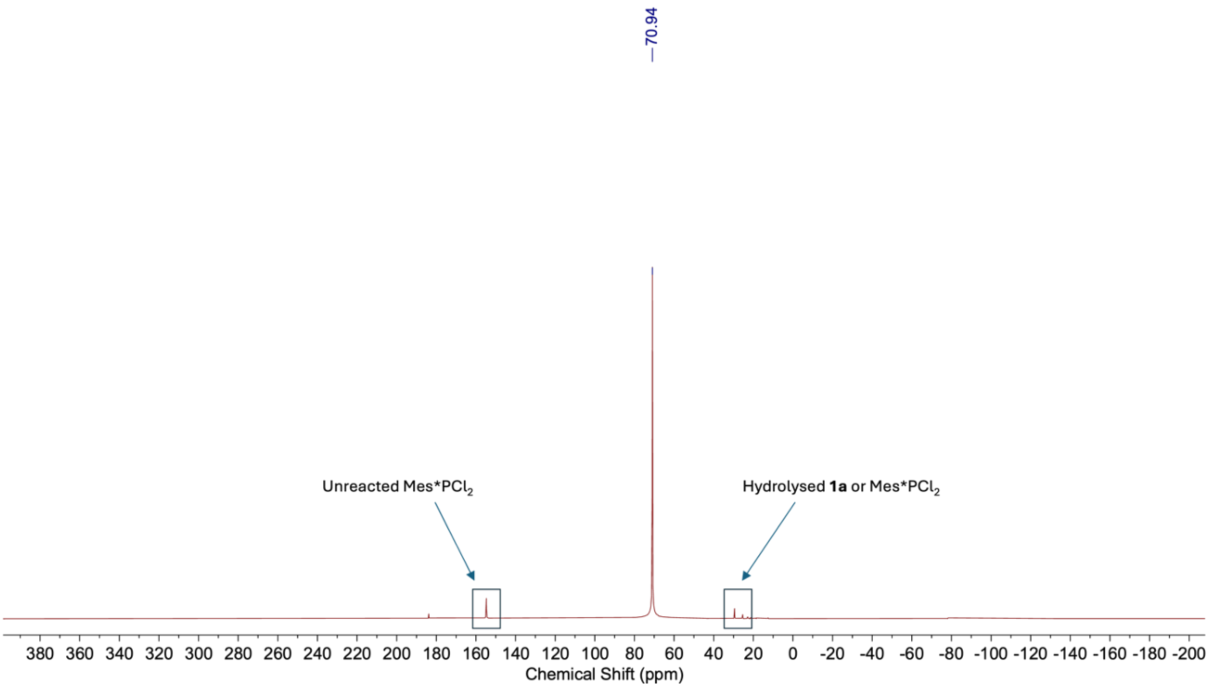

Figure S14. ^{31}P NMR spectrum of intermediate **4a** (crude reaction aliquot). The identification of the impurities is annotated in the figure.

2. Mechanistic Interpretation

While it is not directly possible to conclude the difference between the three compounds based on the sole transient spectra, this information, together with the 1D-PC-EXSY data and the computational analysis, leads to the following interpretation:

1) After light absorption, compound **1** mainly evolves on the excited singlet surface with sub-ps/ps dynamics. This evolution leads to the funneling to the ground state *via* a conical intersection, which leads part of the population to isomerize, as underlined by the 1D-PC-EXSY experiment. Using viscous solvents does not qualitatively change the overall excited state dynamics but slows the overall observed kinetics. No stimulated or spontaneous emission is observed.
2) Compound **2** evolves similarly to compound **1**. However, part of the excited state population evolves to a long-lived triplet state. The transient absorption spectra trace is relatively small in ΔOD units. The molecule isomerizes nonetheless, as confirmed by the 1D-PC-EXSY experiments.
3) Compound **3** forms a long-lived triplet species like compound **2**, but possibly more efficiently, if we assume similar absorptivities for the triplet species coming from the two different molecules. Interestingly, we do not observe relevant signals in the 1D-PC-EXSY which would support the isomerization via a light-triggered mechanism. Vice versa, we observed a small NOE peak corresponding to a magnetization transfer to the solvent (benzene). It is possible that this molecule, which is characterized by low lying charge-transfer states,[6] does not isomerize photochemically. More importantly, it is possible that at the excited state, compound **3** interacts closely with the solvent. We hypothesize that, considering the timescales, this last signal could come from the long-lived triplet of the molecule. Indirectly, with this experiment, we reinforce our hypothesis that isomerization proceeds mainly from a singlet state, while the triplet (more prominent in **3** compared to **2** or **1**) opens up different reactivities.

3. References

[1] Bruker APEX3 V20191-0 St. Version 840A SADABS Version 20161 Bruker AXS Inc Madison Wis. USA **n.d.**

[2] G. M. Sheldrick, Acta Crystallogr. A **2008**, 64, 112–122.

[3] O. V. Dolomanov, L. J. Bourhis, R. J. Gildea, J. a. K. Howard, H. Puschmann, J. Appl. Crystallogr. **2009**, 42, 339–341.

[4] S. Freeman, M. J. P. Harger, J. Chem. Soc. Perkin 1 **1987**, 1399–1406.

[5] Y. V. Svyaschenko, A. Orthaber, S. Ott, *Chem. – Eur. J.* **2016**, *22*, 4247–4255.

[6] A. El Nahhas, M. A. Shameem, P. Chabera, J. Uhlig, A. Orthaber, *Chem. – Eur. J.* **2017**, *23*, 5673–5677.

[7] J. J. Snellenburg, S. Laptenok, R. Seger, K. M. Mullen, I. H. M. van Stokkum, J. Stat. Softw. **2012**, 49, 1–22.

[8] "GitHub - pprcht/crest at 3.0prerelease," can be found under https://github.com/pprcht/crest/tree/3.0prerelease, **n.d.**

[9] P. Pracht, E. Caldeweyher, S. Ehlert, S. Grimme, **2019**, DOI 10.26434/chemrxiv.8326202.v1.

[10] C. Bannwarth, E. Caldeweyher, S. Ehlert, A. Hansen, P. Pracht, J. Seibert, S. Spicher, S. Grimme, WIREs Comput. Mol. Sci. **2021**, 11, e1493.

[11] P. Pracht, C. Bannwarth, J. Phys. Chem. Lett. **2023**, 4440–4448.

[12] S. Lee, M. Filatov, S. Lee, C. H. Choi, J. Chem. Phys. **2018**, 149, 104101.

[13] S. Lee, E. E. Kim, H. Nakata, S. Lee, C. H. Choi, J. Chem. Phys. **2019**, 150, 184111.

[14] G. M. J. Barca, C. Bertoni, L. Carrington, D. Datta, N. De Silva, J. E. Deustua, D. G. Fedorov, J. R. Gour, A. O. Gunina, E. Guidez, T. Harville, S. Irle, J. Ivanic, K. Kowalski, S. S. Leang, H. Li, W. Li, J. J. Lutz, I. Magoulas, J. Mato, V. Mironov, H. Nakata, B. Q. Pham, P. Piecuch, D. Poole, S. R. Pruitt, A. P. Rendell, L. B. Roskop, K. Ruedenberg, T. Sattasathuchana, M. W. Schmidt, J. Shen, L. Slipchenko, M. Sosonkina, V. Sundriyal, A. Tiwari, J. L. Galvez Vallejo, B. Westheimer, M. Włoch, P. Xu, F. Zahariev, M. S. Gordon, J. Chem. Phys. **2020**, 152, 154102.

[15] A. D. Becke, J. Chem. Phys. **1993**, 98, 1372–1377.

[16] R. Ditchfield, W. J. Hehre, J. A. Pople, J. Chem. Phys. **1971**, 54, 724–728.

[17] W. J. Hehre, R. Ditchfield, J. A. Pople, J. Chem. Phys. **1972**, 56, 2257–2261.

[18] P. C. Hariharan, J. A. Pople, Theor. Chim. Acta **1973**, 28, 213–222.

[19] A. D. McLean, G. S. Chandler, J. Chem. Phys. **1980**, 72, 5639–5648.

[20] M. M. Francl, W. J. Pietro, W. J. Hehre, J. S. Binkley, M. S. Gordon, D. J. DeFrees, J. A. Pople, J. Chem. Phys. **1982**, 77, 3654–3665.

[21] J. Steinmetzer, S. Kupfer, S. Gräfe, Int. J. Quantum Chem. **2021**, 121, e26390.

[22] F. Neese, F. Wennmohs, U. Becker, C. Riplinger, J. Chem. Phys. **2020**, 152, 224108.

[23] F. Neese, WIREs Comput. Mol. Sci. **2022**, 12, e1606.

[24] S. Grimme, A. Hansen, S. Ehlert, J.-M. Mewes, J. Chem. Phys. **2021**, 154, 064103.

[25] F. Weigend, R. Ahlrichs, Phys. Chem. Chem. Phys. **2005**, 7, 3297–3305.

[26] V. Ásgeirsson, B. O. Birgisson, R. Bjornsson, U. Becker, F. Neese, C. Riplinger, H. Jónsson, J. Chem. Theory Comput. **2021**, 17, 4929–4945.

[27] D. Casanova, A. I. Krylov, Phys. Chem. Chem. Phys. **2020**, 22, 4326–4342.
